# A new unbiased and highly automated approach to find new prognostic markers in preclinical research

**DOI:** 10.1093/database/baz107

**Published:** 2019-11-18

**Authors:** Martin Neidnicht, Daniela Mittermüller, Katharina Effenberger-Neidnicht

**Affiliations:** Institut für Physiologische Chemie, Universitätsklinikum Essen, Universität Duisburg-Essen, Duisburg 47057, Germany

## Abstract

Data acquisition in (pre)clinical studies is often based on a hypothesis. Numerical algorithms, however, may help to find biomarkers from existing data without formulating any hypothesis. By simply assessing whether a statistical relationship exists between two parameters from a (unlimited) database, every (in)conceivable combination of data becomes a hypothesis. The aim was to create an unbiased and highly automated approach for secondary analysis of (pre)clinical research, including the possibility of a non-linear functional relationship. In our example, an almost homogeneous database was formed by overall 45 parameters (vital, blood and plasma parameters) measured in 11 individual experimental studies at 6 different time points using 57 rats without and 63 rats with systemic inflammation following lipopolysaccharide infusion. For each rat, four group classifiers (treatment, survival, study, ID) were used to get valid samples by a later filtering of the statistical base. Any information about the hypothesis leading to the respective studies was suppressed. In order to assess whether a statistical relationship exists, a total of six different functional prototypes (linear and non-linear) were postulated and examined for their regression. Regression quality, correlation and significance were obtained in form of matrices. In our example, ultimately 510 300 regressions were optimized, automatically evaluated and filtered. The developed algorithm is able to reveal statistical relationships from a nearly crude database with low effort by systematic and unbiased analysis. The finding of well-known correlations proves its reliability, whose validity could be increased by clean aggregation of different studies. In addition, new interesting hints for future research could be gained. Thus, unknown markers can be found which are associated with an increased risk of death during systemic inflammation and sepsis. A further development of the program is planned including multiple regressions (more than two parameters could be related to each other) or cluster analysis.

## Categorization within the Biomedical Field

The use of multivariate statistical methods is important for biomedical research. With regard to the secondary analysis of biomedical data, we have developed an algorithm that is able to reveal new statistical relationships from seemingly exhausted data. For this purpose, several sepsis studies in rats, including all clinical parameters measured, were combined to form the data basis (see Supplementary data): sepsis is defined as a ‘life-threatening organ dysfunction caused by a dysregulated host response to an infection’ (1). In the experimental models that form the basis of this analysis, sepsis was induced in rats by the administration of so-called lipopolysaccharides (LPS), which frequently are found on gram-negative bacteria (1) and therefore can be used to induce a sepsis (2). The treatment of sepsis is still difficult; the mortality is high (30–50%) despite intensive medical care (1). New prognostic biomarkers could enable a faster diagnosis and improve the course of therapy.

## Introduction

The purpose of data mining is to achieve knowledge and to systematically find patterns, trends and information from a huge amount of data (3). Data mining previously was described to be highly beneficial for biomedical research (4). By measuring certain markers/predictors, e.g. in the treatment of diseases, the prognosis could be improved and the therapy adjusted. To date, however, data mining has gained little importance in preclinical research (4).

Data acquisition in (pre)clinical studies is often based on a hypothesis /knowledge. Regardless of whether the hypothesis is confirmed or disproven, the remaining data are often not extensively used and further experiments are planned in which context new data will be generated. This leads to the accumulation of a large amount of unexhausted information. However, such cumulative data harbor undiscovered problems and ideas. An unbiased approach to analyze existing data could reform the planning of further experiments and make them more efficient and less expensive, than blindly creating a new hypothesis. This step could be supported through certain machine learning techniques to automatically recognize statistical connections and favored patterns (4). Here, we focus on finding new possible predictors/biomarkers for sepsis starting form a nearly crude database (formed by 11 individual experimental studies, see Supplementary data) by treating every conceivable and inconceivable combination of data as a potential hypothesis. Any information about the hypothesis leading to the respective studies was suppressed. Since most biomedical processes have a non-linear behavior (e.g. the sigmoid relationship of the oxygen saturation curve (5)), it was important to us to include, in particular, the analysis of non-linear functional relationships that would not be found with traditional correlation. By automating this process of data mining, new correlations occurred which could draw attention to new early and late markers for sepsis.

## Objective

The aim of the project was to define and program a mathematically clean, unbiased and highly automated approach that is specific to the needs for secondary analysis of biomedical research. In particular, this includes the possibility of investigating non-linear functional relationships that would not be found with traditional linear correlation. This is because the assumption that biomedical measurands always change in lockstep is unrealistic and often only due to the limited functionality of the software tools used (6). Furthermore, the statistically prepared results should also be considered as a basis for targeted questions as well as attractive visualization.

**Table 1:** From a crude database to new potential biomarkers for sepsis in six steps. Definition and implementation of group classifiers and populations, functional prototypes and calculation of measures of goodness of fit as well as optimization of the curve fitting and postprocessing

Step 1	The making of a crude database - Collecting of all desirable parameters and as much samples as possible 11 individual studies with 45 clinical parameters (P1–P45)^A^ at six time points ->overall 120 rats						^A^These 45 parameters were measured in all 11 studies. The study design was the same in all studies (see Supplementary data).
Step 2	The making of a statistical base - Rearranging the parameters regarding the group classifiers (G1–G4)^B^ and sample size^C^ G1 (treatment): 63 LPS-treated rats vs. 57 control rats G2 (survival): survivors vs. non-survivors G3 (ID) G4 (study) ->Using the two classifiers ‘treatment’ and ‘survival’, already seven different populations* can be postulated: (i) LPS-treated rats that survived, (ii) LPS-treated rats that did not survive, (iii) all LPS-treated rats, (iv) control rats that survived, (v) control rats that did not survive, (vi) all control rats, (vii) all rats—independent of the treatment						^B^We decided to use these four classifiers to filter for LPS-treatment and survival—to find prognostic markers—but also to filter for the individual studies and rats—to find e.g. seasonal differences or differences between the experimenters. ^C^For later questions and to form clean vectors without missing values, the available sample size was determined for each parameter combination. *Compare also**Figure 1**, A and B.*
Step 3	Linear and non-linear regression^D^ with customized functional prototypes - Combination of the parameters as if every combination is supposed to be the hypothesis - 2025 parameter combinations per time point (one linear functional prototype) - 12 150 parameter combinations for all six time points (one linear functional prototype) - 85 050 parameter combinations for all six time points and seven populations^*^ (one linear functional prototype) - 510 300 parameter combinations for all six time points and seven populations^*^ (one linear and five non-linear functional prototypes) -…^E^						^D^Lavindrasana *et al*. provided a list of data mining methods/function and algorithms found in the literature: classification, clustering, regression, summarization, dependence modeling, visualization and much more (3). Our algorithm is specially tailored to this data set, but easily can be adapted to other data sets at any time. It works without any hypothesis/knowledge and considers both linear and non-linear regressions. Our algorithm generates statistically processed results as a basis for complex questions and appealing visualizations. ^E^If more classifiers or populations are taken into account, you get even more possible combinations.
	Linear regression ${f}_{\mathrm{lin}}=a\ast x+b$	Sigmoid regression ${f}_{\mathrm{sig}}=a+(b-a)\ast \Big(\frac{b\ast c}{c+(b-c)\ast {\exp}^{(-1\ast d\ast b\ast (x-e))}}\Big)$	Exponential regression ${f}_{\mathrm{exp}}=a\ast {\exp}^{(b\ast x+c)+d}$	Quadratic regression ${f}_{\mathrm{quad}}=a\ast {x}^2+b\ast x+c$	Cubic regression ${f}_{\mathrm{cub}}=a\ast {x}^3+b\ast {x}^2+c\ast x+d$	Logarithmic regression ${f}_{\mathrm{log}}=a+\ln (b\ast x+c)+d$	
Step 4	Calculation of measures for goodness of fit - Minimizing the sum-of-squares errors as the objective function for each simulation $\displaystyle\frac{\sum\limits_{i=1}^n{(f({a}_i)-{b}_i)}^2}{\sum\limits_{i=1}^n{(\overline{b}-{b}_i)}^2}=\min$						
Step 5	Optimization of the curve fitting - Numerical optimization using the downhill simplex algorithm^F^ The downhill simplex method (Nelder–Mead method) does not need any prerequisites for the functions to be optimized; in the case of other common optimization methods^G^, these would be e.g. continuity, derivability and convexity. $\nabla \sum\limits_{i=1}^n{(f({a}_i)-{b}_i)}^2\approx 0\forall k\ \mathrm{in}\ f$ Since we cannot be sure that the minimum found is unique, $n$ start estimates in the confidence interval were determined by Monte Carlo approaches for each optimization run.						^F^Programming of numerical algorithms was done in C. ^G^The Newton method (Newton–Raphson method) as well as the SQP (sequential quadratic programming) method, as two examples, are iterative methods for non-linear optimization, but they make certain assumptions about the function to be optimized. *Compare also**Figure 2*.
Step 6	Postprocessing and manual reading - Filtering the optima^H^ A unique search key was assigned to each optimum found, which ensures a secure traceability for later searches.						^H^Visualization was done using MATLAB; calculation was done in Excel. *Compare also**Figures 3**and**4*.

**Figure 1:**
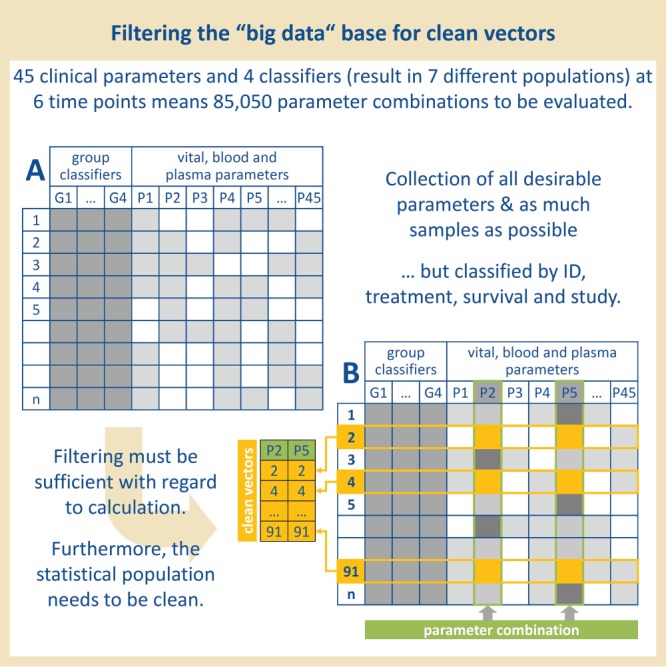
Organization of the data. Previous collected data of 45 clinical parameters measured in overall 120 rats at six time points were selected. The rats were provided with four classifiers to allow filtering according to these classifiers and to ensure a statistical clean population for later questions.

**Figure 2:**
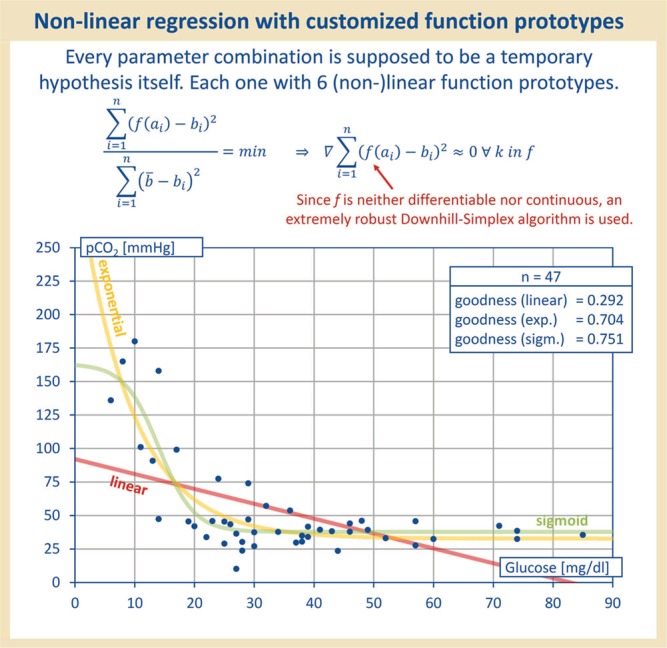
Each parameter combination was treated as hypothesis. To check for any association of parameters, the data were all applied to each other and regression with linear, sigmoid, exponential, quadratic, cubic and logarithmic function prototypes was performed. Therefore, each parameter combination was treated as a hypothesis itself. As shown in this figure, the linear function prototype did not always yield the best correlation value.

**Figure 3:**
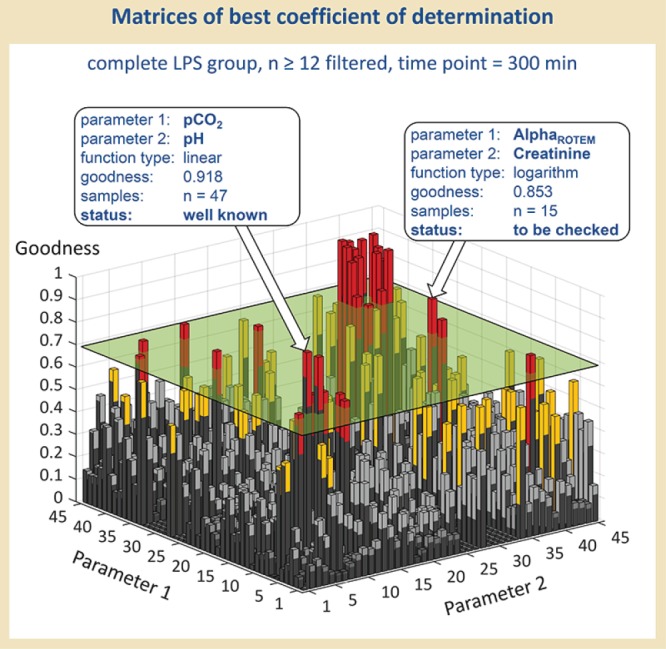
Visualization of the best correlation values. Results were filtered according to a minimal sample size and a minimum value for goodness of fit of the respective parameter combination, and visualized, as an example, as the best-fitting function types for each parameter combination regarding time point 300 min and Population III (means all LPS-treated rats, survivors and non-survivors).

**Figure 4:**
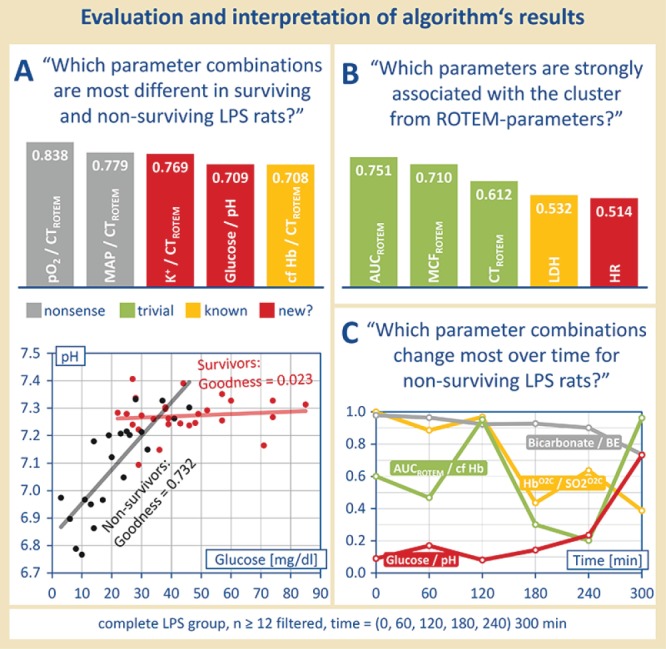
Postprocessing and manual reading. Regression quality, correlation and/or significance matrices can be used to answer complex questions, as formulated in (**A**), (**B**) and (**C)**, in an automated manner. The use of suitable algorithms can reveal e.g. possible clusters of parameters and changes over time.

## Methods

### Step 1: the making of a crude database

The prerequisite for every data mining is the existence of a homogeneous data basis (see Table 1). Our secondary analysis covered data of 45 clinical parameters (P1–45), which were recorded over various time points in several experimental studies (Supplementary data). All parameters were collected in the same weighting: we chose previously collected data covering vital, blood and plasma parameters of overall 120 rats (63 LPS-treated rats = ‘septic’ rats, 57 control rats = ‘non-septic’ rats). The rats received humane care according to the standards of the Federation of European Laboratory Animal Science Association (FELASA). The experimental procedure has been reviewed and approved by the local Animal Care and Use Committee with the permit numbers 84-02.04.2012.A205, 84-02.04.2013.A015, 84-02.04.2014.A357 and 84-02.04.2014.A004. The parameters were collected over the time points 0, 60, 120, 180, 240 and 300 min after LPS treatment in overall 11 studies. The same experiment was done by different experimenters to eliminate execution errors slipped in throughout the studies. By clean aggregation of the different studies, the validity should be increased. Some of the studies have already been published; the list of publications can be found in the Supplementary data.

### Step 2: the making of a statistical base

For each test object, four group classifiers (treatment, survival, study, ID) were used for later sorting and filtering of the statistical base (see Table 1, Figure 1, A): for later questions, we attributed each rat with the classifiers G1–4 which allowed us—for a start—the formation of seven different populations ((i) LPS-treated rats that survived, (ii) LPS-treated rats that did not survive, (iii) all LPS-treated rats, (iv) control rats that survived, (v) control rats that did not survive, (vi) all control rats, (vii) all rats—independent of the treatment). The classifiers ‘ID’ and ‘study’ were less relevant to the formation of these seven populations—however, it could be important concerning the question of seasonal differences or the influence of individual experimenters in further investigations.

In order to generate a structured basis for solid statistical statements from our pure data collection, the available maximum sample size was determined for each parameter combination (see Figure 1, B): only ’valid samples’ with data available for both parameters to be analyzed were stored in two ’clean vectors’.

### Step 3: linear and non-linear regression with customized functional prototypes

To assess whether a statistical correlation exists, a total of six different functional prototypes (linear, logarithmic, sigmoid, quadratic, exponential, cubic) were postulated and examined with regard to their regression (see Table 1): each of the 2025 single parameter combinations (starting from a 45 × 45 matrix) per functional prototype and per time point were treated as hypothesis. Regarding the seven possible populations postulated in Step 2, one gets overall more than 500 000 regressions to be analyzed.

### Steps 4 and 5: calculation of the measures for goodness of fit and optimization of the curve fitting

Since not only linear regression was to be tested—only for linear regression a correlation coefficient is adequate (6)—the sum-of-squares error (SSE) serves as a target function to be minimized, later being the criterion used for the model optimization by the downhill simplex method of Nelder and Mead, which is known to be particularly robust against different radii of convergence and even discontinuities (see Table 1). Thus, the function prototypes, which elude a closed or numerically benevolent treatment, can be reliably optimized. For each parameter combination, the best-fitting function type ultimately was selected. As demonstrated in Figure 2, regression with the linear function prototype did not always yield the best correlation value.

### Step 6: postprocessing and manual reading

The results of all calculations can be understood as regression quality, correlation and/or significance matrices that continuously remain in the working memory and, for example, can be used to a visualization (see Figure 3) or for answering complex questions (see Figure 4). The selection of the interesting matrices can be made dependent onvarious criteria—the last challenge was to relate the different matrices regarding the classifiers (populations) or time points to each other. This process led to the discovery of previously unrecognized correlations, indicating new potential predictors and biomarkers for sepsis.

## Results

Regarding our 45 × 45 matrix, ultimately 510 300 regressions were optimized, automatically evaluated and filtered. Calculation time was about 1 h for each time point. The algorithm suggested several dozen parameter combinations, which were prepared for manual reading to decide whether they are nonsense, trivial, known or even new.

First of all, we filtered data according to a minimum value for goodness of fit and a minimal sample size of the respective parameter combination. As an internal standard, we used 0.7 as the minimum value for goodness of fit and *n* = 12 as minimal sample size to eliminate ‘non-sense’ correlations and to guarantee validity. Figure 3 demonstrates, as an example, the MATLAB visualization of the best-fitting function type for each parameter combination regarding time point 300 min and Population III (means all LPS-treated rats, survivors and non-survivors)—following filtration according to the sample size of 12 and more. Those parameter combinations, which additionally show values for a goodness of fit of 0.7 and more, are lying above the cutting plane and sticking out as columns. These ‘outstanding’ parameter combinations have been further processed in a manual evaluation.

Based on the classifiers postulated in Step 2, several strategies for the determination of suitable sepsis biomarkers have been developed. One problem was to find parameter combinations which were particularly different in surviving and non-surviving LPS rats (see Figure 4A): ‘Which parameter combinations are most different in surviving and non-surviving LPS-rats?’ Therefore, as an example, the difference of values for goodness of fit of each parameter combination in Population I and Population II was formed. Noticeable parameter combinations due to their different course in LPS-treated surviving and deceased rats were cell-free hemoglobin and the clotting time (ROTEM) (see Figure 4A, above), which already was described by Villagra *et al*. (7) as well as Jaegers *et al*. (2). Moreover, also unknown correlations of parameter were found. A correlation of the blood glucose concentration with the pH value should be emphasized here. Surviving LPS-treated rats show a steady pH value wherein the glucose concentration in the blood can vary. On the contrary, non-surviving LPS-treated rats showed a linear correlation of the blood pH value and the blood glucose concentration—the pH value decreased with a decreasing blood glucose concentration (see Figure 4A, below). Consequently, if a low blood pH value occurs together with a low blood glucose concentration, there is a great possibility that the septic rat will not survive. However, this association found is not as trivial as it may seem first.

Even more complex questions like ’Which parameters are strongly associated with the cluster from ROTEM-parameters?’ might be asked. Moreover, the use of suitable sorting algorithms can reveal possible clusters of parameters that are still not visible in the current form (see Figure 4B).

Another approach to find possible markers/predictors for sepsis outcome was to determine the time elapse of parameter correlations (see Figure 4C). Through determining the behavior of all parameter combinations (values for goodness of fit of the best-fitting function type) at different time points, a temporal trend of these parameter combinations was set up, with certain pairs showing a conspicuous course at certain time points. Figure 4C demonstrates, as an example, ‘parameter combinations which changed most over time for non-surviving LPS rats’: after filtering for the sample size (12 and more), the goodness of fit (0.7 and more) and the population (II, non-surviving LPS rats), the values of goodness of fit for the best-fitting function types were obtained for each time point. By using a suitable algorithm, those parameter combinations which changed the most over time can be separated (see Figure 4B).

For example, there are parameters that march in lockstep, such as bicarbonate and BE—which in principle cause each other—showing always a high correlation regardless of the time. However, early biomarkers could be made up, e.g. the parameters of venular blood flow Hb^O2C^ and SO_2_^O2C^ in relation to each other, which initially run in step, but no longer with progressive disease. Furthermore, there are late biomarkers, such as glucose and pH in relation, which initially do not correlate, but progressively more as the disease progresses. Again, other parameter combinations can be assigned neither to the early nor to the late biomarkers, but are still interesting and should be further investigated.

## Discussion

Our algorithm was able to reveal suitable relationships from a nearly crude database with low effort by systematic and unbiased analysis. The finding of well-known correlations proves its reliability, whose validity could be increased by clean aggregation of different studies with similar design.

Moreover, the algorithm was proved as a suitable approach to reveal previously unknown statistical connections, which can be used as targets of further investigations. By assigning classifiers, statistically clean population groups could be formed. Based on these groups, some questions already could be answered without carrying out new experiments, saving time and money. In our case, data mining revealed correlations which we did not notice before, like the correlation of the blood glucose concentration with the blood pH value. Of note, neither a study examining this phenomenon in rats has been published yet nor have contradicting findings been described in another model (8).

This especially highlights the possible role of data mining in (pre)clinical research as an instrument to find new strategies and targets of research. Through data mining, associations can be emphasized—but here, too, correlation does not imply causality. In order to be able to confirm causal relationships, all findings must be investigated experimentally.

## Conclusion

It is possible to read new relationships from seemingly exhausted data by intelligently combining several studies—including all parameters measured. The high degree of automation and the specially tailored software keep the workload for their search low. Even for hypotheses that have already been investigated, a statistically clean combination of different study results increases the sample size and, thus, the validity and significance. Since the calculation methodology has been completely rewritten, it would be, moreover, a good idea to expand the relationship between more than two parameters and perform a multiple regression or cluster analysis.

What is special about our new approach?

1. The algorithm is specially tailored to the data set, but easily can be adapted to other data sets at any time.

2. The algorithm works without any hypothesis/knowledge.

3. The algorithm considers both linear and non-linear regressions.

4. The algorithm generates statistically processed results as a basis for complex questions and appealing visualizations.

## Supplementary Material

Data_mining_MN_DM_KEN-23012019-Supplement_baz107

## Notes

**Database URL:** https://academic.oup.com
