# Supplementary material for: A new unbiased and highly automated approach to find new prognostic markers in preclinical research
Source: Database (Oxford). 2019 Nov 18;2019:baz107. doi: 10.1093/database/baz107 (PMC6859257; doi:10.1093/database/baz107)
Supplement: Data_mining_MN_DM_KEN-23012019-Supplement_baz107 [file data_mining_mn_dm_ken-23012019-supplement_baz107.docx]

**A new unbiased and highly automated approach to find new prognostic markers in preclinical research**

Martin Neidnicht, Daniela Mittermüller, Katharina Effenberger-Neidnicht

Institut für Physiologische Chemie, Universitätsklinikum Essen, Universität Duisburg-Essen, Germany

Address for correspondence:

Dr. rer. nat. Katharina Effenberger-Neidnicht

Institut für Physiologische Chemie

Universitätsklinikum Essen (Universität Duisburg-Essen)

Hufelandstr. 55, D-45147 Essen, Germany

Tel: +49 201 723 4103

E-mail: katharina.effenberger-neidnicht@uni-due.de

Keywords: lipopolysaccharide, sepsis, secondary analysis, predictors and biomarkers, linear and non-linear regression

**Supplement**

**S1. Studies**

|  | **Permit number** | **Publication** | **LPS dose** | **N**  **control** | **N**  **LPS** |
| --- | --- | --- | --- | --- | --- |
| **Study 1** | 84-02.04.2012.A205 | **Effenberger-Neidnicht K**, Jägers J, Verhaegh R, Kirsch M (2018) Therapeutic effects of physostigmine during systemic inflammation. J Inflamm Res; 11: 465. | 0.5 mg LPS/kg | **3** | **5** |
| **Study 2** | 84-02.04.2012.A205 | Brencher L, Oude Lansink M, **Effenberger-Neidnicht K** (2017) Administration of exogenous melatonin after the onset of systemic inflammation is hardly beneficial. Inflammation; 40: 1672. | 0.5 mg LPS/kg | **4** | **8** |
| **Study 3** | 84-02.04.2012.A205 | not published yet | 0.5 mg LPS/kg | **4** | **8** |
| **Study 4** | 84-02.04.2012.A205 | Oude Lansink M, Görlinger K, Hartmann M, de Groot H, **Effenberger-Neidnicht** K (2016) Melatonin does not affect disseminated intravascular coagulation but diminishes decreases in platelet count during subacute endotoxaemia in rats. Thromb Res; 139: 38.  Oude Lansink M, Patyk V, de Groot H†, **Effenberger-Neidnicht K** (2016) Melatonin selectively reduces changes to small intestinal microvasculature during systemic inflammation. J Surg Res; 211: 114. | 0.5 mg LPS/kg | **8** | **12** |
| **Study 5** | 84-02.04.2012.A205 | Preliminary experiments to:  **Effenberger-Neidnicht** K, Jägers J, Verhaegh R, de Groot H (2014) Glycine selectively reduces intestinal injury during endotoxemia. J Surg Res; 192: 592. | 0.5 mg LPS/kg | **4** | **4** |
| **Study 6** | 84-02.04.2012.A205 | **Effenberger-Neidnicht** K, Jägers J, Verhaegh R, de Groot H (2014) Glycine selectively reduces intestinal injury during endotoxemia. J Surg Res; 192: 592. | 0.5 mg LPS/kg | **4** | **8** |
| **Study 7** | 84-02.04.2012.A205 | Preliminary experiments to:  **Effenberger-Neidnicht K**, Brauckmann S, Jägers J, Patyk V, Waack IN, Kirsch M (2018) Protective Effects of Sodium Pyruvate during Systemic Inflammation Limited to the Correction of Metabolic Acidosis. Inflammation; doi: 10.1007/s10753-018-0917-1. | 0.5 mg LPS/kg | **4** | **4** |
| **Study 8** | 84-02.04.2012.A205 | **Effenberger-Neidnicht K**, Brauckmann S, Jägers J, Patyk V, Waack IN, Kirsch M (2018) Protective Effects of Sodium Pyruvate during Systemic Inflammation Limited to the Correction of Metabolic Acidosis. Inflammation; doi: 10.1007/s10753-018-0917-1. | 0.5 mg LPS/kg | **4** | **4** |
| **Study 9** | 84-02.04.2013.A015 | Preliminary experiment to:  Brauckmann S, **Effenberger-Neidnicht** K, de Groot H, Nagel M, Mayer C, Peters J, Hartmann M (2019) Lipopolysaccharide induced hemolysis is abolished by inhibition of thrombin generation but not inhibition of platelet aggregation. Crit Care; submitted. | 0.5 mg LPS/kg | **5** | **5** |
| **Study 10** | 84-02.04.2014.A357 | Brauckmann S, **Effenberger-Neidnicht** K, de Groot H, Nagel M, Mayer C, Peters J, Hartmann M (2015) Lipopolysaccharide-induced hemolysis: Evidence for direct membrane interactions. Sci Rep; 6: 35508. | 0.5 mg LPS/kg | **3** | **5** |
| **Study 11** | 84-02.04.2014.A004 | not published yet | 1 mg LPS/kg | **10** | **-** |
| **Study 12** | 84-02.04.2012.A205 | **Effenberger-Neidnicht K**, Jägers J, Verhaegh R, Kirsch M (2018) Therapeutic effects of physostigmine during systemic inflammation. J Inflamm Res; 11: 465. | 0.5 mg LPS/kg | **4** | **-** |

**S2. Parameters**

| **Number in table** | **Parameter** |
| --- | --- |
| **1** | Weight |
| **2** | Mean arterial blood pressure |
| **3** | Pulse |
| **4** | Respiratory rate |
| **5** | Temperature |
| **6** | pH |
| **7** | CO^2^ partial pressure |
| **8** | O^2^ partial pressure |
| **9** | Base Excess |
| **10** | Bicarbonate |
| **11** | Glucose |
| **12** | Lactate |
| **13** | Hematocrit |
| **14** | K^+^ ion concentration |
| **15** | Na^+^ ion concentration |
| **16** | Ca^2+^ ion concentration |
| **17** | Cl^-^ ion concentration |
| **18** | Hemolysis |
| **19** | Aspartate amino transferase |
| **20** | Alanine amino transferase |
| **21** | Lactate dehydrogenase |
| **22** | Creatine kinase |
| **23** | Creatinine |
| **24** | Urea |
| **25** | Wet dry weight lung |
| **26** | Blood lung |
| **27** | Wet dry weight liver |
| **28** | Blood liver |
| **29** | Wet dry weight kidney |
| **30** | Blood kidney |
| **31** | Wet dry weight heart |
| **32** | Blood Heart |
| **33** | Wet dry weight intestine |
| **34** | Blood intestine |
| **35** | Macroscopic hemorrhage |
| **36** | Maximum clot firmness (ROTEM ®) |
| **37** | Alpha angle (ROTEM ®) |
| **38** | Clotting time (ROTEM ®) |
| **39** | Clot formation time (ROTEM ®) |
| **40** | Area under the curve (ROTEM ®) |
| **41** | Platelet per 100 erythrocytes |
| **42** | Circulation through skeletal muscle |
| **43** | Blood content in skeletal muscle |
| **44** | Oxygen Saturation in skeletal muscle |
| **45** | Nitric oxide content of plasma |
